# Association between HDL Cholesterol Levels and the Consumption of Vitamin A in Metabolically Healthy Obese Lebanese: A Cross-Sectional Study among Adults in Lebanon

**DOI:** 10.1155/2018/8050512

**Published:** 2018-06-03

**Authors:** J. Zalaket, L. Hanna-Wakim, J. Matta

**Affiliations:** Department of Nutrition, Faculty of Agriculture and Food Sciences, Holy Spirit University of Kaslik, Jounieh, Lebanon

## Abstract

**Objectives:**

Previous studies show the association between vitamin A and elevation of plasma triglyceride concentrations. However, limited information exists on the association between vitamin A and plasma HDL cholesterol concentrations. The aim of this study is to investigate the association between plasma HDL cholesterol levels and vitamin A intake in 57 metabolically healthy obese (MHO) Lebanese.

**Methods:**

Out of the 112 adult obese participants who had completed anthropometric and biochemical data, 57 (22 males and 35 females) aged 18–62 years old are metabolically healthy and their data are included in this study. A valid semiquantitative food frequency questionnaire (SQFFQ) was used to test vitamin A intake among other antioxidants. The participants were recruited from the database of three dietary clinics across Lebanon.

**Results:**

Pearson's correlation coefficient was used to measure the strength of the relationship between vitamin A and plasma HDL cholesterol levels. There was a significant positive correlation (*P* value = 0.0225) between vitamin A consumption and HDL cholesterol serum levels in obese participants; when vitamin A levels decrease, HDL levels decrease more in female than in male participants.

**Conclusion:**

The association between dietary vitamin A, a powerful antioxidant, and high HDL levels is shown in MHO but should be further exploited in future studies.

## 1. Background

Dyslipidemia is among the risk factors of cardiovascular disease, which is the most common cause of death worldwide. Dyslipidemia is characterized by a low level of HDL (high-density lipoprotein cholesterol) also known as "good cholesterol" [1]. These observations triggered intense interest in increasing HDL levels for therapeutic intervention of cardiovascular disease because of its antiatherogenic properties [2]. The HDL is a powerful antioxidant itself, more probably an inhibitor of LDL oxidation* in vitro*, and may play an important role* in vivo* in preventing atherosclerosis by inhibiting LDL oxidation in the artery wall [3]. Moreover, metabolic syndrome is associated with greater production of atherogenic lipoprotein particles, and this may alter carotenoid distribution among lipoproteins [4]. Vitamin A, one of the first vitamins to be discovered, is a fat-soluble compound present in many colored vegetables and fruits [5]. The antioxidant activity of vitamin A is conferred by the hydrophobic chain of polyene units that can quench singlet oxygen, neutralize thiyl radicals, and combine with and stabilize peroxyl radicals [6]. Although there is considerable discrepancy in the results from studies in humans regarding the relationship between HDL and vitamin A, we conducted this study to evaluate whether vitamin A intake may exert a protective effect through an impact on high-density lipoprotein serum levels.

## 2. Methods

### 2.1. Participants

112 obese Lebanese were recruited while visiting dietary clinics for routine investigations. Three clinics were involved, two located in rural area in Bekaa governorate and one located in urban area in Kesrouane governorate in Lebanon. Recruitment was done by the same nutritionist who summarized the purpose of this study. Participants were examined between June 2017 and October 2017. They had completed dietary as well as anthropometric and biochemical data in assigned dates, 17 September and 22 October 2017, in two dietary clinics. To be appropriate for participation, participants had to be obese (BMI ≥ 29.9 Kg/m^2^) and age range had to be between 18 and 62 years. They should have no clinical signs of vitamin A deficiency and do not take any nutritional supplements (except vitamin D) or any medications treating high triglycerides, high cholesterol levels, high LDL levels, low HDL levels, diabetes, or hypertension.

This study was approved by the Holy Spirit University Review Board, and all participants signed a written, informed consent.

### 2.2. Metabolic Syndrome

To date, there is no consensus on the MHO phenotype's definition. A commonly used definition is the Adult Treatment Panel criteria (ATP-III) for the metabolic syndrome, which was used in this study. Accordingly, participants with at least three out of five individual factors (increased waist circumference (WC) [according to population/country-specific definition] >94 cm (men) and >80 cm (women) [7]; raised blood pressure (BP) (systolic BP ≥ 130 and/or diastolic BP ≥ 85 mm Hg and/or antihypertensive treatment); hypertriglyceridemia (≥ 150 mg dL); low HDL cholesterol (<40 mg dL or <50 mg dL) in males or females, respectively; and increased fasting glucose ≥100 mg dL and/or antidiabetic treatment) would qualify for metabolic syndrome [8]. 55 participants among 112 who have met at least three out of the NCEP-ATP III MetS criteria at the time of screening were excluded from the study.

### 2.3. Biochemical Analysis

Blood samples were drawn by two trained nurses in the morning after 12-hour overnight fast from antecubital veins and collected into K2EDTA coated collection tubes. Serum was prepared by centrifugation at 2000 g for 15 minutes at 4°C. Aliquots were stored at -20°C until analysis (the next day of collection). Plasma HDL cholesterol and TG were measured simultaneously using an automated clinical chemistry analyzer using enzymatic methods and fasting blood glucose was determined by an oxidase method. The three parameters were measured using Roche Cobas C501 in Medical Care Center laboratory, Zahle.

### 2.4. Lipoprotein Isolation

Lipoprotein fractions were isolated from fasted plasma by sequential ultracentrifugation. This method separates lipoprotein fractions by their differential flotation in fixed solvent densities. Lipoproteins were isolated using an Optima LE-80K ultracentrifuge. After appropriate density adjustments, HDL fractions were sequentially isolated by ultracentrifugation at 200 000 g at 10°C for three hours using a VTi 65.2 rotor in Medical Care Center laboratory, Zahle.

### 2.5. Anthropometric Measures

Participants' body weight was measured by a trained nutritionist, while they wore light clothing and no shoes (digital scale), with an accuracy of 0.1 kg; height measurements (stadiometer) had an accuracy of 1 cm. BMI is calculated by the following equation: BMI (kg/m^2^) = body weight/height^2^. The WC was measured half-way between the lower rib edge and the upper iliac crest by means of a metric measure with accuracy of 0.1 cm. All measures were taken in duplicate and the means of two were used.

### 2.6. Blood Pressure

Seated blood pressure was measured by two trained nurses using a digital sphygmomanometer. Two readings were obtained for both systolic blood pressure and diastolic blood pressure, at 5-minute intervals, and their average was used in this study.

### 2.7. Vitamin A Intake

A semiquantitative food frequency questionnaire (SQFFQ) was used on 57 MHO; it covers the major sources of vitamin A (e.g., sweet potato, veal liver, spinach, carrots, fortified whole milk, mango, cowpeas, apricots, and broccoli). The development, validity, and reproducibility of this questionnaire are studied elsewhere. Dietary intake from the SQFFQ was transformed into daily intake of each food (g/d) and beverage (ml/d) by multiplying the specific portion unit by the frequency of consumption using the following values for reported frequencies: 1–3 times/d = 2 ((1+2+3)/3); 1–3 times/w = 0.28 ((1/7+2/7+3/7)/3); 1–3 times/month = 0.06 ((1/30+2/30+3/30)/3); occasionally and never eaten = 0.

### 2.8. Statistical Analyses

Descriptive statistics for anthropometric and biochemical characteristics of MHO were presented as means ± SD. Pearson's correlation coefficient was used to measure the strength of the relationship between vitamin A and plasma HDL cholesterol levels. Student's unpaired* t*-test was used to compare the differences between males and females in different parameters, age, education levels, BMI, fasting blood glucose, triglycerides and HDL cholesterol levels, WC, blood pressure, and vitamin A intake. All analyses were undertaken using the statistical software package SPSS® (Statistical Package for Social Sciences, version 24.0, SPSS Inc., Chicago, IL, USA). Results with *P* < 0.05 value will be considered statistically significant.

## 3. Results

Among 112 obese Lebanese volunteers (40 males and 72 females), 57 were metabolically healthy (51%).  Table 1 showed the characteristics of the study participants. Age, BMI, BP, glucose levels, triglycerides levels, and education level were not significantly different between participants (*P* > 0.05). However, WC was significantly higher in men than in women (*P* < 0.05) and serum HDL levels were significantly higher in women than in men (*P* < 0.05).

The mean of vitamin A consumption is lower in women (776.9 ± 172.9 mg RAE) than in men (918.7 ± 192.3 mg RAE). This difference is significant with *P* value = 0.02.

Pearson's correlation coefficient was used to measure the strength of the relationship between vitamin A consumption and serum HDL levels. There was a significant positive correlation (*P* value = 0.0225) between vitamin A consumption and the serum levels of HDL cholesterol in obese participants; when vitamin A levels decrease, HDL levels decrease more in female than in male participants (*P* value =0.0084).

## 4. Discussion

Obesity is a principal causative factor in the development of metabolic syndrome. Increased oxidative stress in accumulated fat is an important pathogenic mechanism of obesity-associated metabolic syndrome [9], which is a constellation of risk factors characterized by central obesity, dyslipidemia, hypertension, and hyperglycemia associated with insulin resistance [10]. Consequently, obesity is generally associated with numerous cardiometabolic disorders. However, studies have shown that a subset of obese individuals exist, which do not display such cardiometabolic abnormalities (metabolically healthy) and were identified under an obese phenotype termed MHO [11].

Moreover, obesity has become one of the primary health concerns in many parts of the world because of its increasing contribution to the burden of global morbidity [12]. The Middle East, in particular, faces the greatest threat in terms of the growing obesity epidemic [13]. For example, in Lebanon, a developing and middle-income country, the prevalence of obesity has reached alarming rates among adults (20 years and older) [14]. It increased from 17.4% in 1997 to 28.2% in 2009 (27.4% for men and 28.8% for women in 2009) [15]. Such a worrying trend underscores the need to examine the proportions of obese individuals who are metabolically healthy and to investigate associated dietary factors with metabolic parameters. In this study, 57 participants among 112 were considered MHO (51%); this percentage is higher than 37.2% MHO and overweight participants in a recent study done on a Lebanese population by Matta* et al.* in 2016.

Moreover, the results of the current study demonstrate that the age, BMI, blood pressure, glucose levels, and triglycerides levels were not significantly different between participants (*P* > 0.05). However, WC was significantly higher in men than in women (*P* < 0.05); the mean of WC in men was 104.4 ± 12.4 cm versus 88.8 ± 10.4 cm in women, which was compatible with the WHO cutoffs [7] for Europid population (>94 cm in men and >80 cm in women). Likewise, serum HDL levels were significantly lower in men (40.2 ± 6 mg/dL) than in women (52.9 ± 12.9 mg/dL) (*P* < 0.05), which is compatible with other studies, where normal HDL levels should be >40 mg/dL in men and >50 mg/dL in women [16].

According to an analysis of data from the 2007-2008 National Health and Nutrition Examination Survey (NHANES), the mean of vitamin A consumption was 776.9 ± 172.9 mg RAE, higher than the average dietary vitamin A intake in Americans aged 2 years and above (607 mcg RAE). Moreover, the mean of dietary vitamin A was significantly lower (*P* < 0.05) in Lebanese adult women (776.9 ± 172.9 mg RAE) than in men (918.7 ± 192.3 mg RAE). Similar to what has been reported in other populations, American adult men have slightly higher intakes (649 mcg RAE) than adult women (580 mcg RAE). Although these intakes are lower than the RDAs for individual men (900 mg RAE) and women (700 mg RAE), these intake levels were considered adequate for population groups [17].

Furthermore, a high intake of vitamin A has been associated with a decreased risk for cardiovascular disease [18]. We conducted this study to evaluate whether vitamin A intake may exert a protective effect through an impact on high-density lipoprotein serum levels. There was a significant positive correlation (*P* value = 0.0225) between vitamin A consumption and the serum levels of HDL cholesterol in obese participants; when dietary vitamin A levels decrease, serum HDL levels decrease more in female than in male participants (*P* value = 0.0084). Information is scarce regarding this association; however, dietary carotenoids (precursor of retinol, the active form of vitamin A) are a group of nearly 600 compounds, and only about 50 of these have provitamin A activity [6]; they share intestinal absorptive pathways with dietary cholesterol [4]. Furthermore, similar to dietary cholesterol, there are considerable interindividual responses in plasma carotenoids to dietary changes, attributed to reduced dietary intake of vitamin A and obesity [4]. Once in the body, the metabolic fate of vitamin A is determined in part by the route of its plasma carrier. Lipoproteins are the major carriers of vitamin A in circulation, with polar carotenoids (xanthophylls) primarily transported on HDL particles [4]. Also, dietary modifications aimed at increasing plasma lipoprotein concentrations of lutein and zeaxanthin (provitamins A) may help prevent the development of chronic diseases associated with metabolic syndrome. They also reported an inverse association of BMI or abdominal obesity and adipose tissue concentrations of carotenoids due to reduced dietary consumption and modifications of lipoprotein carriers (e.g., reduced HDL-C) [4].

The strengths of this study are the following: we chose the MHO sample to work with because metabolic syndrome is associated with greater production of atherogenic lipoprotein particles, and this may alter HDL levels [4]. Also, the sample was representative of the Lebanese population at large, because the project was concentrated in rural and urban areas.

But the findings of this study ought to be considered within the context of its limitations. Ideally, our results should be validated in study populations with larger numbers of MHO participants, and body fat percentages as a criterion for correct classification of obesity should be considered. Furthermore, beta-carotene intake should be considered along with vitamin A intakes and cofounding factors should be taken into consideration. It should be noted that more studies should be done to clarify the association between dietary vitamin A and serum HDL levels, because low levels of HDL might reflect reduced antioxidant protection against LDL oxidation [19] and thus may increase the risk of cardiovascular disease. Such understanding could, in turn, facilitate the development of new treatment and prevention strategies that more effectively reduce the burden of chronic diseases resulting from obesity.

## 5. Conclusions

Therapeutic potential of dietary vitamin A, a powerful antioxidant, in correcting human obesity-associated abnormal lipoprotein metabolism should be exploited after establishing the mechanism through which vitamin A is associated with HDL concentrations. More work, especially concerning the relevance of how tissue concentrations and plasma levels rather than dietary intake relate to HDL levels, is required. The present results should be considered as primary and additional follow-up studies are needed to confirm those findings. This study may represent an important message to healthcare professionals, especially in a population at high risk for cardiovascular disease.

## Figures and Tables

**Table 1:** Descriptive characteristics of the study population.

	**MHO (** **n** ** = 57)**	**Males (** **n** ** = 18)**	**Females (** **n** ** = 39)**	**P** ** value** ^*∗*^
**Age (years)**	38 ± 12.0	34 ± 12.2	39 ± 11.7	0.14

**BMI (Kg/m** ^**2**^ **)**	33.3 ± 3.4	33.44 ± 3.4	33.2 ± 3.2	0.81

**Education**				0.4
**Middle school**	48	12	29	
**High school**	13	4	9	
**University and higher**	3	2	1	

**WC (cm)**	93.7 ± 13.1	104.4 ± 12.4	88.8 ± 10.4	0.03

**Glucose (mg/dL)**	91.9 ± 7.6	91.8 ± 5.2	91.9 ± 8.5	0.96

**Serum triglycerides (mg/dL) **	115.9 ± 50.7	122.1 ± 53.6	113.1 ± 49.8	0.54

**Serum HDL cholesterol (mg/dL)**	48.9 ± 12.7	40.2 ± 6	52.9 ± 12.9	0.01

**Systolic BP (mmHg)**	121 ± 18.1	127 ± 19.4	118.2 ± 16.9	0.09

**Diastolic BP (mmHg)**	78.9 ± 11.1	75.8 ± 10.6	80.4 ± 11.2	0.14

**Vitamin A (mg RAE)**	776.9 ± 172.9	918.7 ± 192.3	711.58 ± 116.72	0.02

(i) Values are expressed as the means ± SD.

(ii) *P* values were derived from independent-sample *t*-test for continuous variables.

## Data Availability

Raw data were generated at Holy Spirit University of Kaslik. Derived data supporting the findings of this study are available from the corresponding author (J. Zalaket) upon request.
